# Mucosal regulatory and effector T-cell imbalance in CVID-associated enteropathy

**DOI:** 10.3389/fimmu.2026.1937502

**Published:** 2026-09-03

**Authors:** Semra Demir, Nevzat Kahveci, Metin Yusuf Gelmez, Derya Unal, Osman Ozan Yegit, Mehmet Akif Yagli, Zulal İstemihan, Neslihan Berker, Bilger Cavus, Fatih Besisik, Esin Cetin Aktas, Asli Gelincik, Gunnur Deniz

**Affiliations:** 1 Division of Immunology and Allergy Diseases, Department of Internal Medicine, Istanbul Faculty of Medicine, Istanbul University, Istanbul, Türkiye; 2 Department of Immunology, Aziz Sancar Institute of Experimental Medicine, Istanbul University, Istanbul, Türkiye; 3 Division of Gastroenterology and Hepatology, Department of Internal Medicine, Istanbul Faculty of Medicine, Istanbul University, Istanbul, Türkiye; 4 Department of Pathology, Istanbul Faculty of Medicine, Istanbul University, Istanbul, Türkiye

**Keywords:** common variable immune deficiency (CVID), effector T cell, enteropathy, intestinal T cells, mucosal immunity, T cell, T cell subgroup, Treg - regulatory T cell

## Abstract

**Background:**

Common variable immunodeficiency (CVID) frequently presents with gastrointestinal complications, yet the immunopathogenesis of CVID-associated enteropathy remains poorly understood.

**Objective:**

This study aimed to investigate intestinal T cell subset distributions and intracellular cytokine profiles in order to better understand mucosal immune alterations associated with enteropathy in CVID.

**Methods:**

Duodenal and ileal biopsy specimens were obtained from CVID patients with enteropathy (EP, n=11) and without enteropathy (EN, n=13), patients with Crohn’s disease (CD, n=9), and healthy controls (HC, n=14). Lamina propria mononuclear cells were isolated and analyzed by flow cytometry to determine T-cell phenotypes, maturation status, and intracellular cytokine production following stimulation.

**Results:**

Duodenal tissue from EP patients showed significantly reduced frequencies of total T, cytotoxic T, mucosal T, mucosal cytotoxic T, accompanied by an expansion of γδ T cells and a trend toward reduced Treg frequencies. Intracellular IL-17 production was markedly decreased in both mucosal and γδ T cell subsets. In the ileum, EP patients displayed reduced Treg frequencies together with increased γδ T cells and enhanced IFN-γ production by γδ T cells.

**Conclusions:**

This study identifies distinct, compartment-specific alterations in duodenal and ileal T cell profiles in CVID-associated enteropathy. Reduced IL-17 production and γδ T cell expansion in the duodenum, together with reduced Treg frequencies and increased IFN-γ-producing γδ T cells in the ileum, characterized CVID-associated enteropathy. These findings indicate disrupted mucosal immune homeostasis and provide a basis for future longitudinal and mechanistic studies.

## Introduction

Common variable immunodeficiency (CVID) is the most common symptomatic inborn error of immunity (1). CVID patients are prone to developing recurrent infectious diseases and non-infectious complications such as lymphoproliferation, autoimmune disorders, allergy, malignancy, or gastrointestinal diseases (2). These complications also result in sequelae, like structural damage and progressive organ dysfunction (3). With advances in immunoglobulin replacement therapy (IgRT) for these patients, infections and their complications can be prevented, thereby substantially reducing associated morbidity and mortality (2, 4). However, IgRT does not prevent non-infectious complications and their sequelae which remain associated with increased morbidity, mortality and significant burden on patients, families and healthcare systems (5, 6).

Although CVID has been characterized by defective B-cell differentiation and hypogammaglobulinemia, evidences suggest that abnormalities in T cell subsets, including altered CD4/CD8 ratios, reduced regulatory T (Treg) cells, and impaired Tcell function, may contribute to immune dysregulation and the development of non-infectious complications; however, data on the precise role of T cell pathology remain limited (7, 8). In patients with CVID, enteric complications, which represent the second leading cause of morbidity following respiratory tract involvement, pose significant challenges for the patient management (9). Enteric involvement in CVID is associated with up to a fourfold increase in mortality and histopathologic abnormalities in up to 58% of upper and 68% of lower gastrointestinal endoscopic samples (4, 10, 11). Enteric complications, also referred to as CVID enteropathy, are not uniformly defined in these patients, particularly regarding whether the diagnosis should be based on clinical symptoms and/or histopathologic findings (12). In some reports, infectious pathologies are excluded from the definition of CVID enteropathy, which appears reasonable (12). Furthermore, there is limited information regarding the immunological mechanisms underlying enteropathy. These factors limit the development of effective and reliable treatment options.

The lack of understanding of the underlying mechanisms of non-infectious enteric complications along with limitations in accurate and effective management, underscores the need for further studies. Therefore, this study aimed to characterize intestinal T cell phenotypes and intracellular cytokine profiles in patients with CVID to elucidate the immunological mechanisms underlying enteropathy.

## Methods

### Study population and design

**Figure 1:**
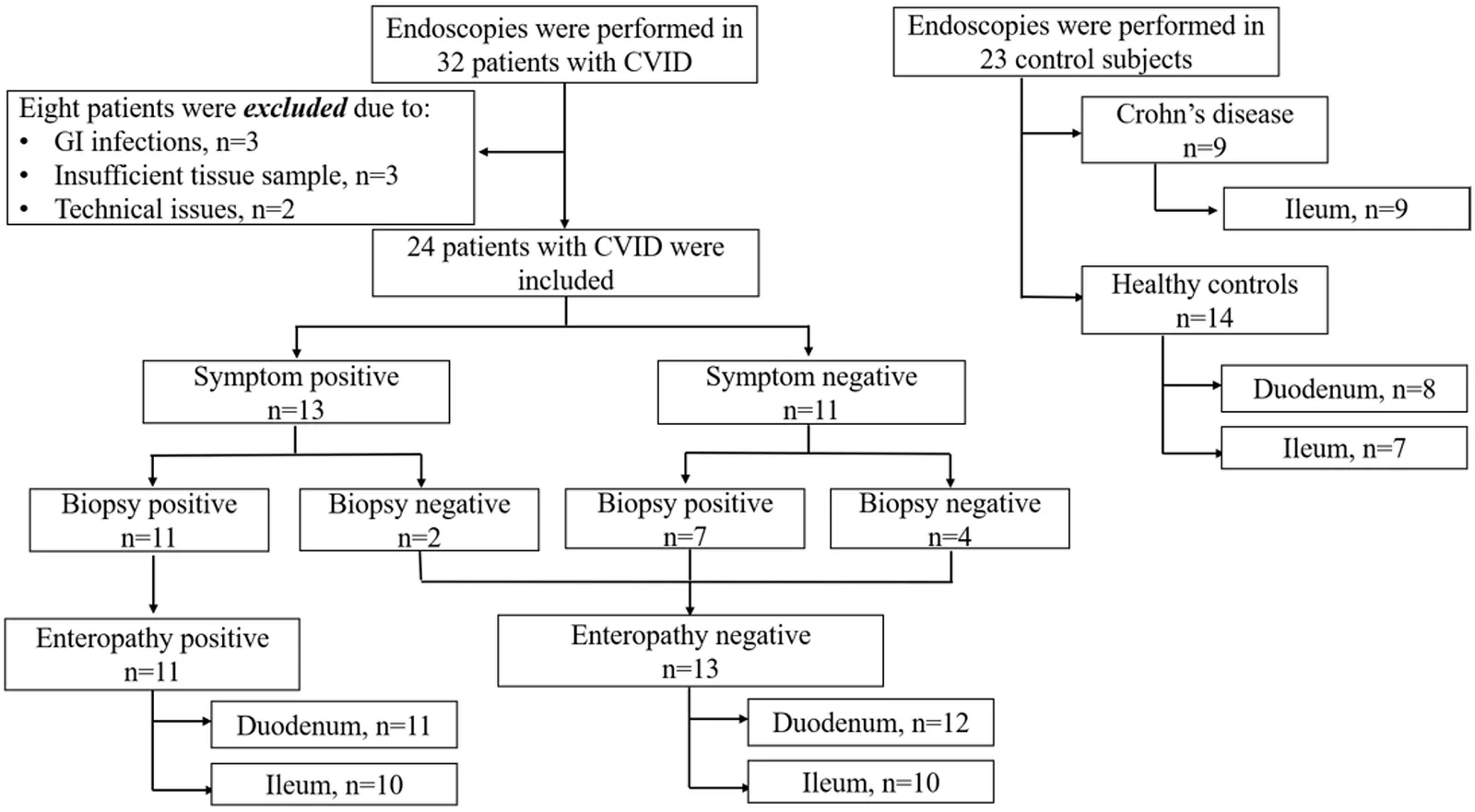
Overview of the study cohorts.

The study included three main groups: patients with CVID, patients with Crohn’s disease and healthy controls. Patients with CVID were classified as enteropathy positive and enteropathy negative based on the presence of both gastrointestinal tract related symptoms and histopathologic evidence of inflammation. The participants underwent upper and/or lower gastrointestinal endoscopy based on indications established by an immunologist and/or gastroenterologist. Adult individuals who underwent endoscopy for check-up or polyp surveillance and showed no histopathological evidence of any enteric disease were included as healthy controls. Patients with CVID or Crohn’s disease were excluded if they were under 18 years of age; had infectious enteritis and were receiving immunosuppressive or immunomodulatory therapy. Patients with Crohn’s disease were included only if they met the diagnostic criteria and had active disease according to the Harvey-Bradshaw Index (13, 14). An overview of the study cohorts is shown in **Figure 1**.

CVID patients were diagnosed according to the European Society for Immunodeficiencies criteria (15). In these patients, demographic and clinical characteristics including age, sex, body mass index (BMI), age at symptom onset, age at diagnosis, duration of follow-up, presence of chronic diarrhea, weight loss, micronutrient deficiency, malnutrition, malabsorption, required IgRT dosage (g/kg/week), and macroscopic and microscopic gastrointestinal findings were evaluated. Biochemical parameters at the time of diagnosis and at the time of endoscopy, and peripheral blood total T cells, CD4^+^ T cells, naïve CD4^+^ T cells, CD8^+^ T cells, CD19^+^ B cells, and class-switched memory B cell values, when available, were obtained from the medical records of patients with CVID. Patients were further evaluated according to whether their proportion of class-switched memory B cells was below or above 2% (16). Chronic diarrhea was defined as frequent loose or watery stools persisting for three months or longer in the absence of an identifiable infectious cause. Malabsorption was defined as an unintended loss of ≥5% of body weight over a 6–12-month period, accompanied by objectively documented nutritional deficiencies. Malnutrition was defined as a condition in which, in addition to malabsorption, parenteral nutritional support is required to maintain a BMI above 18.5 (17).

### Isolation of lamina propria mononuclear cells and cell stimulation

Eight to ten tissue samples (1–3 mm in diameter) were obtained from the most inflamed areas of the duodenum and/or ileum during gastrointestinal endoscopy in patients, whereas samples were obtained randomly from healthy control and from patients without any inflammatory findings, in addition to routine histopathological sampling, for T lymphocyte phenotyping and intracellular cytokine analysis.

Lamina propria mononuclear cells (LPMCs) were isolated from the tissue samples according to a previously published protocol (18). LPMCs were stimulated with phorbol 12-myristate 13-acetate, ionomycin, and Brefeldin A (Leukocyte Activation Cocktail with GolgiPlug™, BD Pharmingen, San Diego, USA) under sterile conditions for 4 h at 37 °C prior to analysis.

### Cell surface and intracellular cytokine staining

After stimulation, LPMCs were stained with anti-human CD3-Alexa Fluor 700, CD4-BV570, CD8-APC-Cy7, CD103-BV421, CD27-BV510, TCRγδ-FITC, CD25-PE, CD45RA-PE-Cy5 monoclonal antibodies (BD Biosciences, San Jose, USA). The samples were vortexed and incubated at room temperature for 15 minutes. Following incubation, cells were washed with FACSFlow sheath fluid (BD Biosciences, San Jose, USA) and centrifuged under the same conditions. For the intracellular staining FixA and FixB solution from the fixation kit (Fix&Perm™ Cell Permeabilization Kit, Thermo Fisher Scientific, USA) and fluorochrome-conjugated antibodies including FoxP3-APC, IL-17A-BV650, IL-4-BV711, IFN-γ-BV786, IL-10-PECY7 (BD Biosciences, San Jose, USA) and IL-12-PerCP-eFluor710 (Thermo Fisher Scientific, USA) for intracellular marker and cytokine staining were added. The samples finally resuspended in FACS Flow Sheath fluid (BD Biosciences, San Jose, USA) for flow cytometric analysis.

### Flow cytometric analysis and gating strategy

The prepared samples were analyzed using flow cytometry. Different lymphocyte subpopulations including total T cells (CD3^+^), cytotoxic T cells (T_C_, CD3^+^CD8^+^), helper T cells (T_H_, CD3^+^CD4^+^), mucosal T cells (CD3^+^CD103^+^), cytotoxic mucosal T cells (CD3^+^CD103^+^CD8^+^), helper mucosal T cells (CD3^+^CD103^+^CD4^+^), gamma delta T cells (γδ T, CD3^+^TCRγδ^+^), Treg cells (CD3^+^CD4^+^CD25^high^FoxP3^+^) were identified on the NovoCyte instrument (ACEA Biosciences, USA) using the NovoExpress software (Agilent Technologies, USA).

Lymphocytes gating strategy is given in **Supplementary Figure 1**. Moreover, the maturation profiles of total and mucosal T cells were determined according to their staining patterns with anti-CD27 and anti-CD45RA antibodies, classifying them as naïve, effector memory (T_EM_), central memory (T_CM_), or terminally differentiated T cells (T_EMRA_) (**Supplementary Figure 2**).

The levels of IL-12, IL-17A, IL-4, IFN-γ, and IL-10 in T_H_, T_C_, mucosal T, mucosal T_C_, mucosal T_H_, and γδ T cells, and the IL-10 expression ratios in Treg cells, were analyzed using the NovoExpress software (**Supplementary Figures 3**, **4**).

Analyses were performed using all evaluable samples for each cell population. In three enteropathy positive samples, γδ T cell frequencies could not be reliably quantified because the TCRγδ staining did not yield sufficient viable γδ T cell events for reliable quantification. Therefore, these samples were excluded only from the γδ T cell analysis.

The study protocol was approved by the Ethics Committee of Istanbul Faculty of Medicine. All participants were informed about the study and its procedures and provided written informed consent.

### Statistical analyses

Data were analyzed using IBM SPSS Statistics software, version 24.0 (Armonk, NY, IBM Corp., USA). Graphical representations were performed using GraphPad Prism version 10 (GraphPad Software, San Diego, CA, USA). Categorical variables were presented as frequencies and percentages (n, %), while continuous variables were expressed as median (interquartile range, IQR). Due to the small number of participants in each group, non-parametric tests were employed. Comparisons of continuous variables among multiple groups were performed using the Kruskal–Walli’s test. Significance levels were adjusted using the Bonferroni correction for *post-hoc* pairwise comparisons following significant Kruskal–Wallis analyses. Comparisons between two groups were carried out using the Mann–Whitney U test. Categorical variables were compared between groups using the chi-square or Fisher’s exact test. A p-value of <0.05 was considered statistically significant.

## Results

### Study population

At the beginning of the study, upper and/or lower gastrointestinal endoscopy was performed in 32 patients with CVID. Eight of these patients were excluded due to reasons such as infection, insufficient tissue samples, or technical issues. Although infectious causes were initially ruled out through stool analyses, two patients with negative stool test results were later found to have giardiasis and one was diagnosed with tuberculosis; these patients were subsequently excluded from the study. CVID patients were further grouped as enteropathy positive (n=11) and enteropathy negative (n=13) (**Figure 1**). The demographic characteristics of participants are presented in **Table 1**.

**Table 1:** Age and gender distribution of participants.

Characteristics	EP n=11	EN n=13	CD n=9	HC n=14	p
Age, years, median (IQR)	43.0 (40-46)	46.0 (42-52)	44.0 (43-54)	48.5 (40-57)	0.429
Gender, F/M	3/8	8/5	4/4	9/5	0.248

EP, Enteropathy positive; EN, Enteropathy negative; CD, Crohn’s disease; HC, Healthy control; IQR, Interquartile range; F, Female; M, Male.

### Clinical and laboratory features of patients with CVID

Comparison of the characteristics of patients with enteropathy (n=11) versus without enteropathy (n=13) is presented in detail in **Table 2**. Enteropathy positive group required significantly higher weekly IgRT doses (p<0.001). As expected, chronic or recurrent diarrhea and micronutrient deficiency were significantly more common in the enteropathy positive group compared to the enteropathy negative group (p=0.046 and p=0.016, respectively). The micronutrient deficiencies identified were vitamin B12 (n=5), vitamin D (n=4), iron (n=3) and folic acid (n=1).

**Table 2:** Clinical and laboratory characteristics of the patients with CVID.

Characteristics	EP n=11	EN n=13	p
Age at symptoms of onset (years), median (IQR)	18 (12.5-30)	28 (10-39)	0.416
Age at diagnosis (years), median (IQR)	35 (26.5-40.5)	40 (36-49)	0.132
CVID related complications, n (%)			
Recurrent sinopulmonary infections	9 (81.8)	13 (100)	0.199
Recurrent gastrointestinal infections	8 (72.7)	7 (53.8)	0.423
Autoimmune diseases	5 (45.5)	5 (38.5)	0.685
Benign lymphoproliferation	6 (54.5)	7 (53.8)	0.768
Allergic diseases	4 (36.4)	3 (23.1)	0.650
Malignancy	2 (18.2)	0	0.158
Bronchiectasis	1 (9.1)	5 (38.5)	0.179
Splenomegaly	4 (36.4)	4 (30.8)	0.685
Chronic/recurrent diarrhea, n (%)	**5/9 (55.6)**	**1/12 (8.3)**	**0.046**
Weight loss, n (%)	2/9 (22.2)	1/12 (8.3)	0.553
Micronutrient deficiency, n (%)	**6/9 (66.7)**	**1/12 (8.3)**	**0.016**
Malabsorption, n (%)	2/9 (22.2)	0	0.171
Required Ig replacement dose (g/kg/week), median (IQR)	**0.2 (0.15-0.2)**	**0.11 (0.1-0.125)**	**<0.001**
BMI (kg/m^2^), median (IQR)	25.23 (21.95-29.2)	25.15 (20-30.5)	0.897
Prophylactic antibiotic usage, n (%)	1 (9.1)	3 (27.3)	0.591
Laboratory findings at the diagnosis, median (IQR)			
IgG (mg/dl)	134 (40-252)	362 (111-472)	0.107
IgM (mg/dl)	16 (5-24)	14.5 (4.5-61)	0.976
IgA (mg/dl)	3 (1-5)	2.5 (0.5-8)	0.879
Albumin (g/dl)	4.7 (4.03-4.7)	4.7 (4.45-4.9)	0.109
Ferritin (ng/ml)	55 (41.5-63.55)	20 (9.75-48.7)	0.200
Vitamin B12 (pg/ml)	**323 (292-354)**	**553 (394-619)**	**0.043**
Absolute lymphocyte count/µL	1950 (1600-2100)	2000 (1400-2800)	0.872
Lymphocyte subsets at the diagnosis, (%), median (IQR)			
Total T cells	**83.4 (81.7-85)**	**69.22 (65.4-75.8)**	**0.017**
T_C_ cells	49.4 (47.35-52.85)	43.6 (34.3-48.7)	0.117
T_H_ cells	37.7 (34.9-43.01)	42 (40.82-47.46)	0.667
Naïve T_H_ cells	23.11 (17.67-23.4)	12.7 (10.2-27.3)	0.833
Total B cells	9.29 (7.5-10.5)	7.73 (6.3-10.4)	0.710
Switched memory B cells	13.92 (12.55-15.29)	7.05 (2.68-11.66)	0.183

Data regarding chronic/recurrent diarrhea, weight loss, micronutrient deficiency, and malabsorption were available for 9 of 11 patients in the EP group and 12 of 13 patients in the EN group. Percentages were calculated using the available data as the denominator. The percentages were calculated using the number of patients with available data for each variable. EP, Enteropathy positive; EN, Enteropathy negative; Ig, Immunoglobulin; IQR, Interquartile range; T_C_, Cytotoxic T; T_H_, Helper T, BMI: Body mass index. Statistically significant values are shown in bold.

At the time of CVID diagnosis, the vitamin B12 levels were significantly lower in the enteropathy positive group compared to the enteropathy negative group (p=0.043). In addition, the percentage of total T cells was higher in enteropathy positive patients than in enteropathy negative patients (p=0.017) (**Table 2**).

The histopathologic analyses of the samples and the endoscopic findings are presented in detail in **Table 3**. Duodenal nodularity and chronic active duodenitis were significantly more frequent in the enteropathy positive group than in the enteropathy negative group (p=0.022 and p=0.037, respectively). In the analysis of the ileal findings, nodularity was more frequently observed in the enteropathy positive group compared to the enteropathy negative group (p=0.025).

**Table 3:** Endoscopic and histopathologic findings in patients with CVID.

Findings	EP	EN	p
Duodenal sampling	n=11	n=12	
Macroscopic, n (%)			
Nodularity	6 (54.5)	1 (8.3)	0.022
Aphthous ulcers	1 (9.1)	0	0.478
Microscopic, n (%)			
Active chronic duodenitis	11 (100)	7 (58.3)	0.037
Lymphoid hyperplasia	7 (63.6)	5 (41.7)	0.414
Villous atrophy	1 (9.1)	0	0.478
Intraepithelial lymphocytosis	1 (9.1)	1 (8.3)	0.949
Chronic inflammatory mucosa with eosinophilic infiltration	5 (45.5)	2 (16.7)	0.130
Lymphangiectasis	2 (18.2)	1 (8.3)	0.571
Foveolar metaplasia	1 (9.1)	3 (16.7)	0.586
Ileal sampling	n=10	n=10	
Macroscopic, n (%)			
Nodularity	7 (70)	2 (20)	0.025
Aphthous ulcers	2 (20)	0	0.474
Microscopic, n (%)			
Active chronic ileitis	8 (80)	4 (40)	0.170
Chronic inflammatory mucosa with eosinophilic infiltration	4 (40)	2 (20)	0.628
Lymphoid hyperplasia	2 (20)	2 (20)	0.709
Cryptitis	2 (20)	3 (30)	0.605
Crypt abscess	1 (10)	0	0.230

EP, Enteropathy positive; EN, Enteropathy negative.

### Duodenal T cell profile analysis

**Figure 2:**
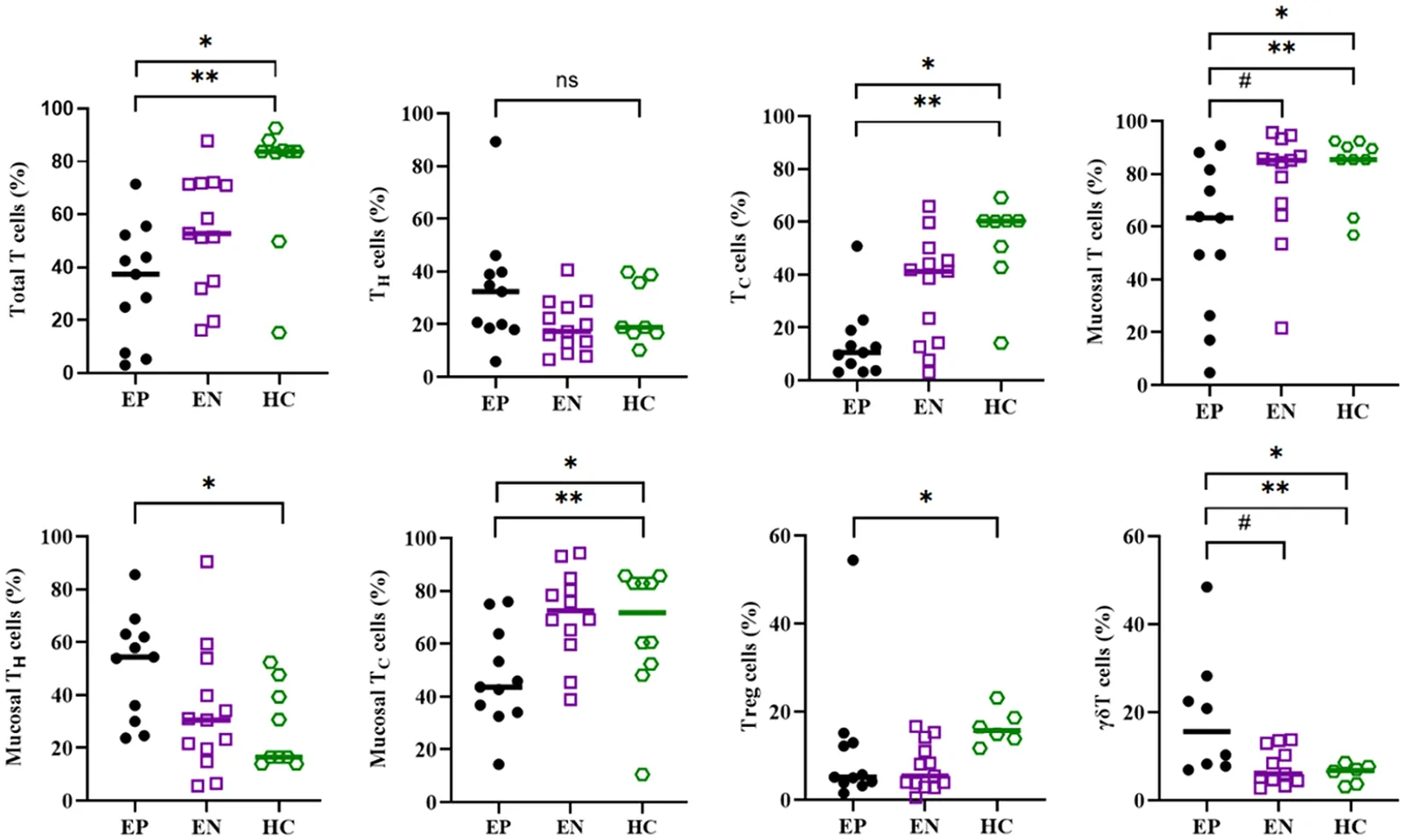
Comparison of duodenal T cell subsets among EP, EN, and HC groups. Individual data points represent participants and horizontal lines indicate medians. Statistical comparisons were performed using the Kruskal–Wallis test followed by *post hoc* pairwise comparisons when appropriate. Symbols indicate the following comparisons: * all groups; ** EP vs HC; # EP vs EN. Significant differences were detected in total T cells (p*=0.005, p**=0.006), T_C_ cells (p*=0.001, p=0.002), Treg cells (p*=0.025), mucosal T cells (p*=0.002, p**=0.010, p#=0.011), mucosal T_H_ (p*=0.002, p**=0.058), mucosal T_C_ (p=0.016, p**=0.049), and γδ T cells (p*=0.007, p**=0.042, p#=0.037) among the study groups. p values were given only for statistically significant comparisons. Sample sizes are indicated for each analysis. In the EP group, γδ T cell data were available for eight participants, whereas the analyses of the other T-cell subsets included all evaluable samples (n=11). Percentages were calculated relative to the following parent populations: total T cells as a percentage of lymphocytes; T_H_, T_C_, γδ T, and mucosal T cells as percentages of total CD3^+^ T cells; mucosal T_H_ and mucosal T_C_ cells as percentages of CD3^+^CD103^+^ mucosal T cells; and Treg cells as a percentage of CD3^+^CD4^+^ T cells. EP, Enteropathy positive; EN, Enteropathy negative; HC, Healthy control; T_H_, Helper T; Tc, Cytotoxic T; Treg, Regulatory T ns, not significant.

Duodenal T cell profiling revealed significant differences among the enteropathy positive, enteropathy negative, and healthy control groups in total T, T_C_, Tregs, γδ T, mucosal T, mucosal T_H_ and mucosal T_C_ cells (p=0.003, p=0.001, p=0.025, p=0.007, p=0.002, p=0.022, p=0.001, respectively). In *post-hoc* analyses, enteropathy positive patients exhibited significantly reduced CD3^+^ T cells and CD8^+^ T_C_ cells compared to the healthy control group (p=0.006, p=0.001). Although Treg cell percentages were lower in both enteropathy positive and enteropathy negative patients compared to the healthy control group, these differences did not reach statistical significance in *post-hoc* analyses. Enteropathy positive patients also showed higher γδ T cell levels compared to both the healthy control group and enteropathy negative patients (p=0.042, p=0.037). Mucosal T cell levels were significantly lower in enteropathy positive patients compared to both enteropathy negative patients and the healthy control group (p=0.011, p=0.010). Additionally, mucosal T_C_ cell levels were reduced in enteropathy positive patients compared to the healthy control group (p=0.049) (**Figure 2**).

**Figure 3:**
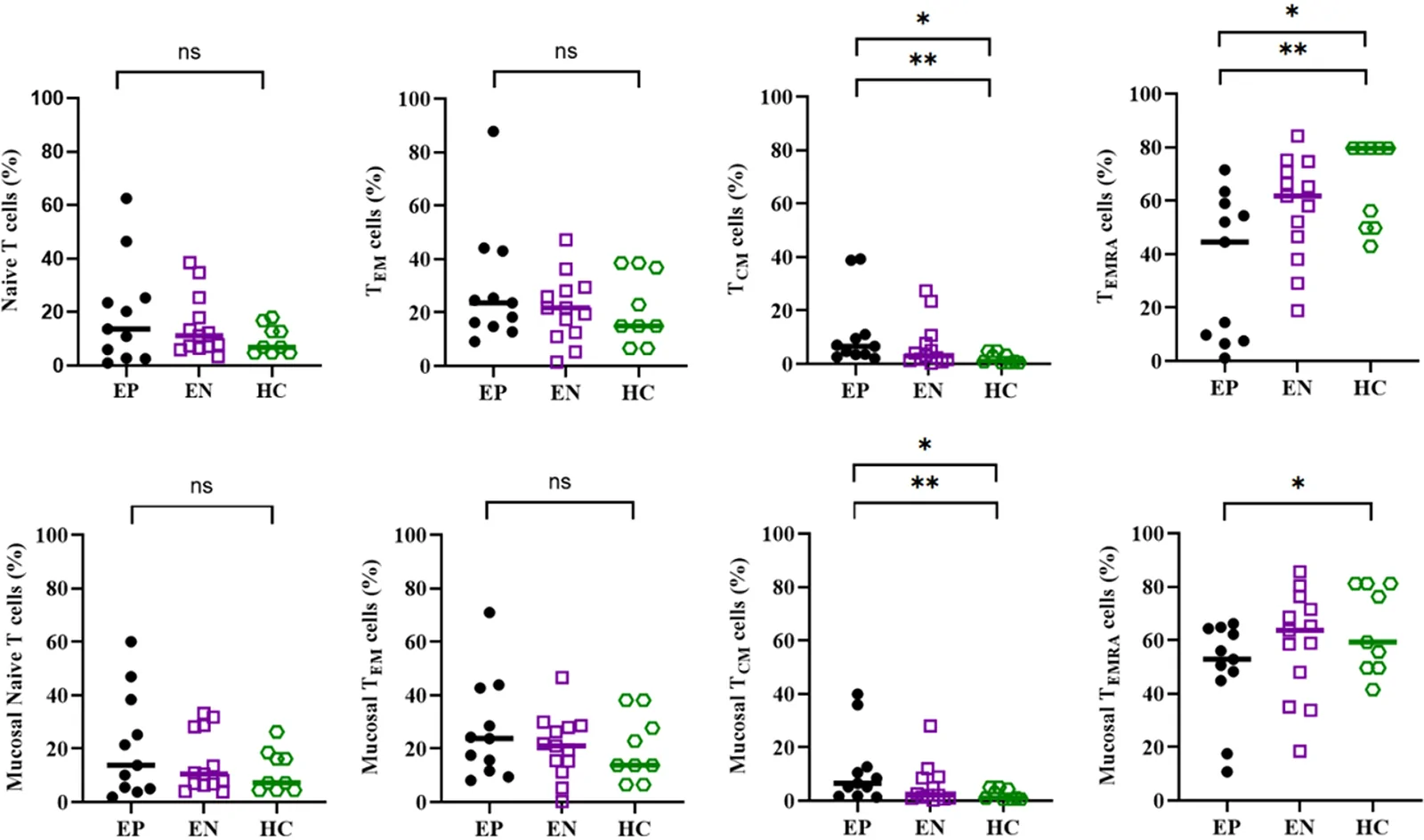
Comparison of maturation profiles of intestinal T and mucosal T cells among groups. Symbols indicate the following comparisons: * all groups; ** EP vs HC. Significant differences were detected in T_CM_ (p*=0.021, p**=0.033), T_EMRA_ (p*=0.025, p**=0.05), mucosal T_CM_ (p*=0.026, p**=0.05) and mucosal T_EMRA_ (p*=0.040) among the study groups. p values were given only for statistically significant comparisons. Naïve, T_CM_, T_EM_, and T_EMRA_ frequencies were calculated as percentages of the total T cell population. Mucosal naïve, T_CM_, T_EM_, and T_EMRA_ frequencies were calculated as percentages of CD3+CD103+ mucosal T cells. EP, Enteropathy positive; EN, Enteropathy negative; HC, Healthy control; T_EMRA_, Terminally differentiated T; T_EM_, Effector memory T; T_CM_, Central memory T ns, not significant.

When comparing the maturation profiles of intestinal T and mucosal T cells across the three groups, the proportions of T_EMRA_ cells, T_CM_ cells, mucosal T_EMRA_ cells, and mucosal T_CM_ cells differed significantly among three groups (p=0.025, p=0.021, p=0.040 and p=0.026, respectively). In *post-hoc* analyses, T_EMRA_ levels were lower in the enteropathy positive group compared with the healthy control group (p=0.05). T_CM_ levels were higher in enteropathy positive patients compared with healthy controls (p=0.033). Although mucosal T_EMRA_ levels showed overall differences among the groups, pairwise *post-hoc* comparisons did not demonstrate significant differences. Mucosal T_CM_ levels were higher in enteropathy positive patients compared with healthy controls (p=0.05) (**Figure 3**).

### Duodenal intracellular cytokine analysis

**Figure 4:**
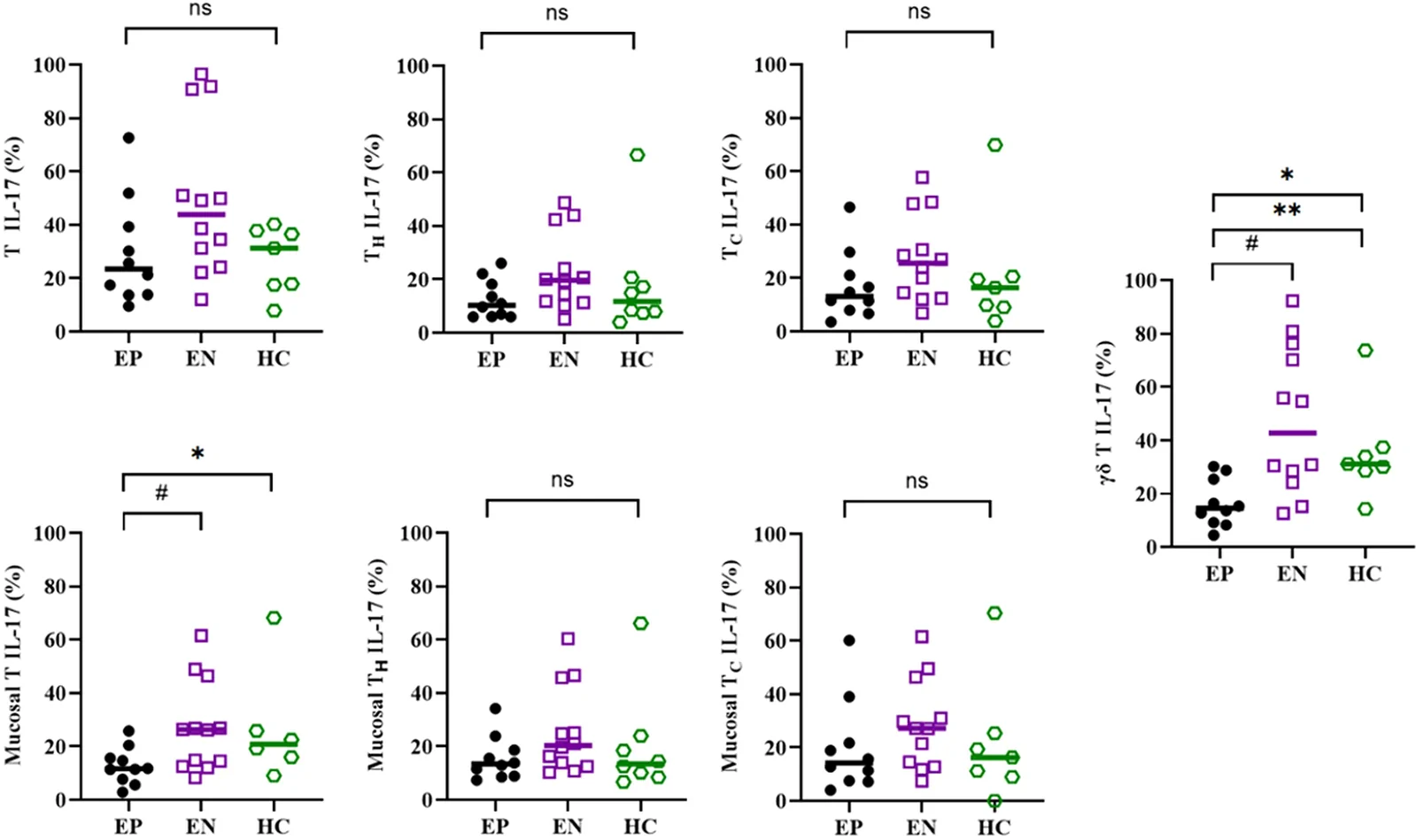
Comparison of duodenal intracellular IL-17 levels among the three groups with *post-hoc* analysis. Symbols indicate the following comparisons: * all three groups; ** EP vs HC; # EP vs EN. Significant differences were detected in IL-17 levels of mucosal T cells (p*=0.007, p**=0.05, p^#^=0.031) and of γδ T cells (p*=0.001, p**=0.032, p^#^=0.03) among the study groups. p values were given only for statistically significant comparisons. For IL-17 analysis, the percentage shown represents IL-17-positive cells within the corresponding gated T cell subset. IL-10-expressing Treg cells were calculated as a percentage of CD3+CD4+CD25highFoxP3+ cells. EP, Enteropathy positive, EN, Enteropathy negative, HC, Healthy control, T_H_, Helper T, Tc, Cytotoxic T ns, not significant.

Intracellular levels of IFN-γ, IL-12, IL-17A, IL-4 and IL-10 was analyzed in stimulated duodenal T, T_H_, T_C_, mucosal T, mucosal T_H_, mucosal T_C_ and γδ T cells. IFN-γ, IL-12, IL-4 and IL-10 levels across all cell types were similar among the three groups (**Supplementary Table 1**). Although IL-17 levels were lower in enteropathy positive patients across all cell subsets, the reduction reached statistical significance only in the mucosal T and γδ T cell populations (p=0.007 and p=0.001, respectively). Pairwise *post-hoc* analyses revealed that mucosal T cell IL-17 levels were lower in enteropathy positive patients compared with enteropathy negative patients (p=0.031). IL-17 levels of γδ T cells were lower in enteropathy positive patients than in both enteropathy negative patients and the healthy controls (p=0.03 and p=0.032, respectively) (**Figure 4**).

### Ileal T cell profile analysis

**Figure 5:**
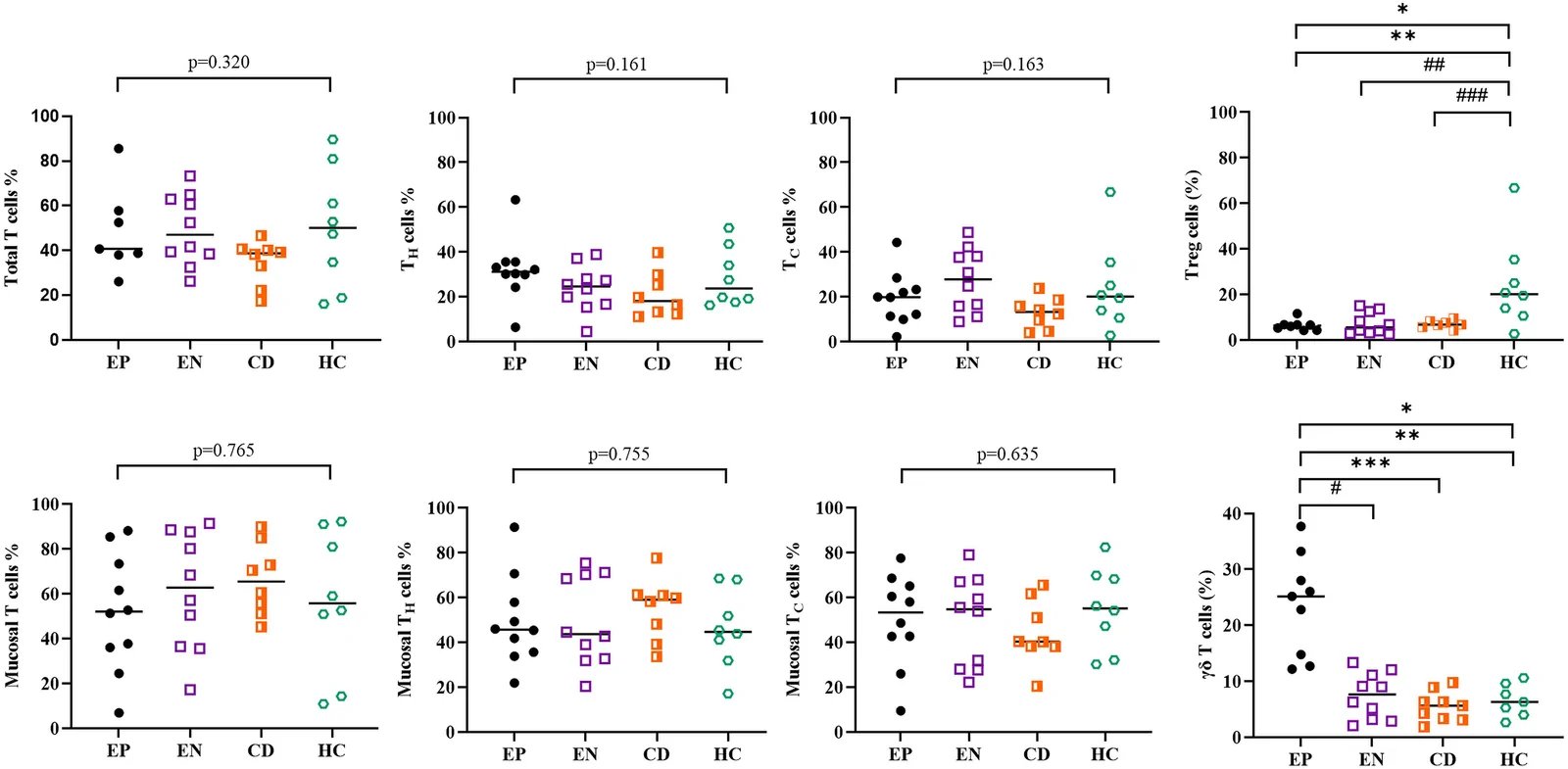
Comparative analysis of ileal intestinal T cell subsets among CVID patients with enteropathy (EP) and without enteropathy (EN), patients with Crohn’s disease (CD) and healthy controls (HC). Symbols indicate the following comparisons: * all four groups; ** EP vs HC; *** EP vs CD; ^#^ *EP vs EN;* ^##^ *EN vs HC;* ^###^ *CD vs HC.* Significant differences were identified in Treg cells (p*=0.001, p**=0.003, p^##^=0.002, p^###^=0.021) and γδ T cells (p*<0.001, p**=0.006, p***=0.001, p^#^=0.006) among the study groups. p values were given only for statistically significant comparisons. Percentages were calculated using the same parent populations as in the duodenal analyses: total T cells relative to lymphocytes; T_H_, T_C_, γδ T, and mucosal T cells relative to CD3^+^ T cells; mucosal T_H_ and mucosal T_C_ cells relative to CD3^+^CD103^+^ mucosal T cells; and Treg cells relative to CD3^+^CD4^+^ T cells. EP, Enteropathy positive; EN, Enteropathy negative; CD, Crohn’s disease; HC, Healthy control; Treg, Regulatory T.

Levels of γδ T cells and Tregs differed significantly across four groups (p<0.001 and p=0.001, respectively). In *post-hoc* analyses, γδ T cells were significantly higher in enteropathy positive patients compared with enteropathy negative patients (p=0.006), patients with Crohn’s disease (p=0.001), and the healthy controls (p=0.006). Treg frequencies were lower in enteropathy positive, enteropathy negative, and Crohn’s disease patients compared to the healthy controls (p=0.003, p=0.002 and p=0.021, respectively) (**Figure 5**). No significant differences were observed in the maturation profiles of ileal T cell subsets among the four groups.

### Ileal intracellular cytokines analysis

**Figure 6:**
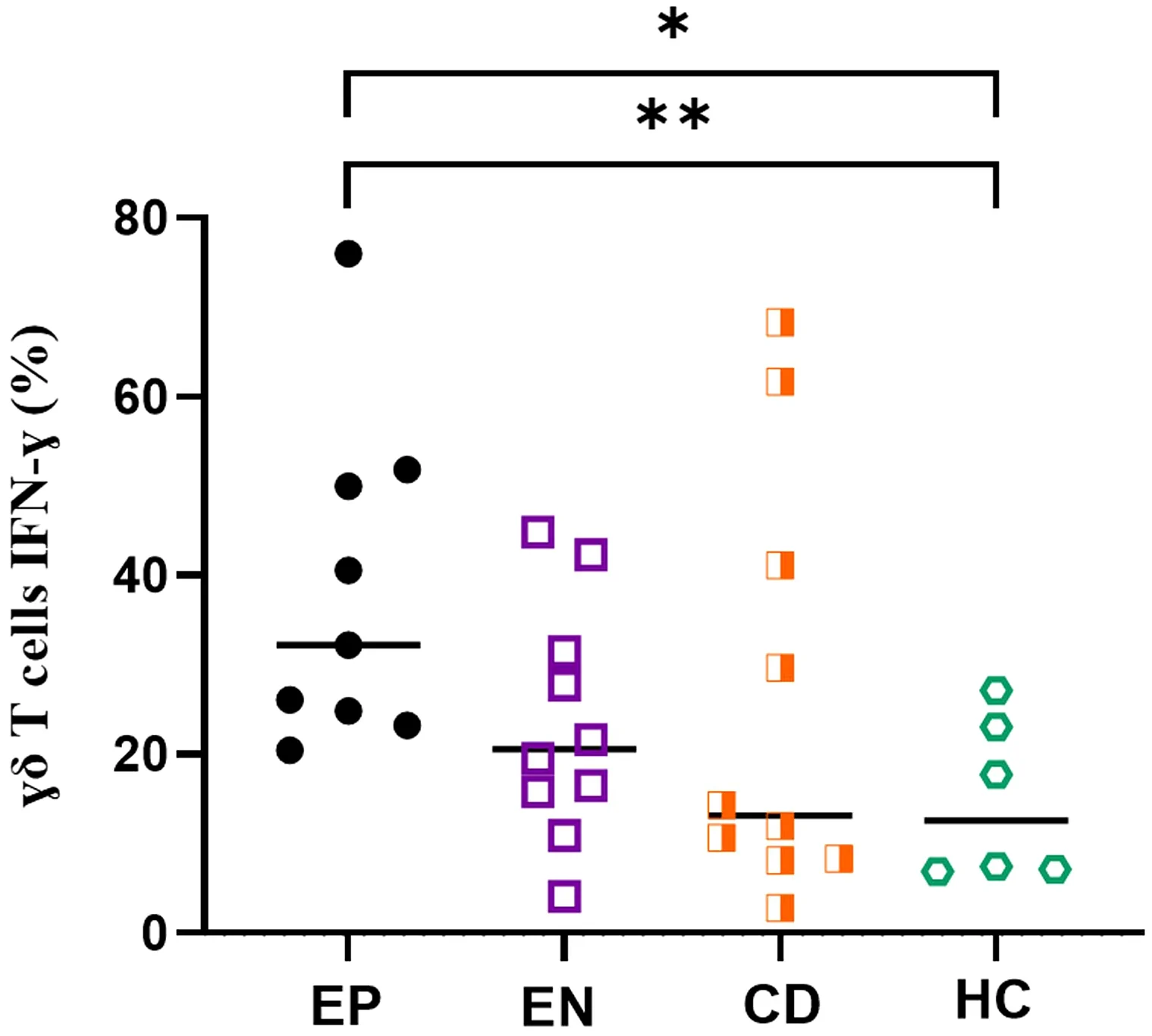
Intracellular IFN-γ expression in ileal γδ T cells in patients with CVID with enteropathy (EP), patients with CVID without enteropathy (EN), patients with Crohn’s disease (CD) and healthy controls (HC). A significant overall difference was detected among the four groups (Kruskal–Wallis test, p=0.041). *Post-hoc* analysis showed higher IFN-γ expression in the EP group than in the HC group (p=0.027). Symbols indicate the following comparisons: * overall comparison among all four groups; ** EP versus HC.

IFN-γ levels in γδ T cells differed significantly among the groups (p=0.041). In pairwise comparisons, IFN-γ levels of γδ T cells were higher in the enteropathy positive group than in the healthy controls (p=0.027) (**Figure 6**).

## Discussion

The mechanisms driving intestinal tissue inflammation are still not well-defined in patients with CVID. To enlighten this problem, we applied an original and previously unreported experimental approach: by selecting patient groups matched for age and gender and isolating LPMCs from duodenal and ileal biopsies, we evaluated T cell subsets and intracellular cytokine profiles by flow cytometry. This approach may enable to understand how immune regulatory defects manifest locally within the gastrointestinal mucosa in CVID. We anticipate that detailed local cellular and cytokine profiling may provide novel insights into the gastrointestinal immunopathogenesis of CVID and may potentially elucidate future targeted therapeutic strategies for CVID associated enteropathy.

The gastrointestinal tract plays a crucial role not only in nutrient absorption but also in systemic metabolic homeostasis. Therefore, intestinal involvement in CVID may lead to metabolic consequences, including alterations in micronutrient levels and immunoglobulin utilization requirements (19). In the USIDNET retrospective registry of CVID patients in the America, those with gastrointestinal disease showed a higher prevalence of vitamin B12 deficiency (20). Similarly, in our study, micronutrient deficiencies were more common, and baseline vitamin B12 levels were lower in patients with enteropathy than in those without gastrointestinal involvement. In a long-term comparison of patients receiving IgRT, those with enteropathy required higher Ig dosages (21). Consistent with these findings, our study demonstrated that IgRT dose requirements were higher in patients with enteropathy than in those without. This finding supports the concept that increased intestinal inflammation may necessitate higher or more frequent IgG administration. Taken together, these observations emphasize the importance of early and systematic nutritional assessment, close monitoring of micronutrient status, and careful adjustment of IgRT regimens in CVID patients with enteropathy. Such strategies may reduce morbidity and improve quality of life in this subgroup of patients. Furthermore, these findings highlight the importance of elucidating the mechanisms underlying enteric complications in CVID.

Despite increasing recognition of its clinical relevance, the mechanisms underlying the development of enteropathy in CVID and the factors determining its associated clinical risks have not yet been fully elucidated. Although multifactorial pathogenesis involving microbial dysbiosis, defective immune regulation, immune deficiency, and autoimmunity has been proposed, data regarding tissue-level immunological alterations in CVID-associated enteropathy remain limited (12). Examination of intestinal immunity reveals that multiple mechanisms contribute to mucosal homeostasis. For example, Treg cells play a central role in maintaining intestinal homeostasis by suppressing aberrant immune responses to enteric antigens, interacting directly with other immune cells, and secreting anti-inflammatory cytokines such as TGF-β and IL-10 (22). In the presence of TGF-β, T lymphocytes express CD103 (integrin αEβ7), acquire a tissue-resident phenotype and contribute to both local defense and inflammatory responses (23). γδ T cells, which colonize mucosal tissues, participate in distinct protective mechanisms. IL-17–producing γδ T cells provide protection against pathogens and contribute to intestinal homeostasis by influencing microbiome composition and exerting regulatory functions. Immunoregulatory γδ T cells further facilitate CD4^+^ T cell polarization, exert immunosuppressive effects, and participate in tissue repair processes (24).

A study involving gene signature analyses of duodenal biopsy samples suggested a mechanism characterized by T_H_-mediated and microbiome-associated IFN-driven inflammation together with reduced mucosal IgA levels (25). Studies in inflammatory bowel disease (IBD) indicate that intestinal inflammation is primarily mediated by CD4^+^ T cells, based on both animal models and cytokine expression patterns in the intestinal mucosa (26). Additionally, autoreactive CD8^+^ T cells have been proposed to initiate disease by targeting epithelial cells, followed by exposure to luminal antigens driving CD4^+^ T cell expansion and exacerbating inflammation (27). Furthermore, Mannon et al., using cytokine analyses rather than cell-based assays, demonstrated reduced IL-17 in stimulated duodenal LPMCs from patients with CVID. They also reported increased IL-12 and IFN-γ levels in patients with enteropathy (25). Our study demonstrated that CVID patients with enteropathy exhibited marked alterations in duodenal T cell composition and cytokine production compared to both CVID patients without enteropathy and healthy controls. Specifically, these patients showed lower total T, T_C_, Treg, mucosal T and mucosal T_C_ cell counts, increased γδ T cell frequencies, and a shift toward a higher proportion of T_CM_ and mucosal T_CM_ cells. Moreover, intracellular IL-17 production was significantly reduced in mucosal and γδ T cell subsets, although IL-17 levels were generally lower across all T cell subsets compared to the other groups. Our findings suggest that IL-17–mediated mechanisms, particularly those driven by mucosal T cells and γδ T cells, may be suppressed in patients with enteropathy, indicating a dysregulated duodenal mucosal immune response. Accordingly, reduced IL-17 production by mucosal and γδ T cells in the enteropathy-positive group may reflect impaired mechanisms involved in mucosal barrier repair and defense against microorganisms. This impairment may contribute to increased intestinal permeability and a higher risk of translocation of pathogenic or commensal microorganisms. Moreover, reduced IL-17 responses may impair epithelial regeneration processes following injury, including tight junction renewal and antimicrobial defense. In this context, our findings support the possibility that altered IL-17 responses contribute to the pathogenesis of enteropathy in CVID. Future studies exploring these mechanisms and developing strategies aimed at restoring or enhancing barrier integrity may hold significant therapeutic potential.

In IBD, Smids et al. (18) reported that patients with Crohn’s disease exhibited decreased mucosal T cells and increased Treg cells in the lamina propria compared to healthy controls. Although duodenal samples from Crohn’s disease patients were not included in our study, and therefore no direct comparison could be made, our finding of reduced Treg cell frequencies in duodenal tissue may support the notion that enteropathy in CVID differs immunologically from classical IBDs. Patients with Crohn’s disease were included primarily as an inflammatory disease control for ileal immune profiling. However, direct comparisons should be interpreted cautiously due to differences in tissue localization and disease pathogenesis.

Another notable finding of our study was the altered maturation profile of duodenal T cells in the enteropathy positive group. Increased T_CM_ together with decreased T_EMRA_ frequencies suggested a shift toward a less terminally differentiated memory T cell phenotype within the duodenal mucosa. T_CM_ cells have substantial proliferative and self-renewal capacity and can generate effector cells following antigenic restimulation; therefore, their accumulation may reflect persistent antigenic exposure and the maintenance of a readily responsive inflammatory T cell pool (28). Previous studies of intestinal inflammatory diseases have similarly demonstrated altered mucosal T cell maturation profiles, including increased T_CM_ frequencies in active IBD, with partial normalization following mucosal healing (18). Conversely, the reduced T_EMRA_ frequency observed in our enteropathy positive group may indicate altered terminal effector differentiation or redistribution of these cells. Nevertheless, intestinal T cell maturation patterns vary according to disease type, tissue compartment, and inflammatory activity. Thus, our findings should be interpreted as an enteropathy-associated shift in T cell differentiation rather than as evidence of a causal mechanism.

In the ileal sample analyses, the proportion of Treg cells decreased, whereas γδ T cell frequencies increased in patients with enteropathy. Consistent with this increase, IFN-γ production by γδ T cells was elevated in the enteropathy positive group relative to healthy controls, while remaining comparable to the other patient groups. Elevated IFN-γ production by ileal γδ T cells may exert pathological effects by disrupting barrier integrity or perpetuating chronic inflammation. Particularly when combined with the reduced Treg cell frequencies in the ileum, this imbalance may further contribute to the loss of mucosal homeostasis. The simultaneous expansion of γδ T cells together with reduced IL-17 and increased IFN-γ production may reflect functional skewing of mucosal γδ T cells toward a more pro-inflammatory phenotype in CVID-associated enteropathy. To date, ileal immune profiles in patients with CVID have not been investigated.

The concurrent decrease in duodenal and ileal Treg cells further suggests a failure to maintain local homeostatic balance within the intestinal environment.

The more limited ileal alterations observed in the current study may suggest regional heterogeneity of mucosal immune dysregulation in CVID-associated enteropathy. Although the more extensive alterations observed in the duodenum could reflect greater inflammatory activity in this compartment, segment-specific disease activity was not systematically assessed; therefore, this interpretation remains speculative. To our knowledge, no previous studies have directly compared detailed duodenal and ileal T cell profiles in patients with CVID-associated enteropathy, making it difficult to interpret these compartment-specific findings in the context of previous research.

Interestingly, the increased peripheral blood T cell frequency in the enteropathy positive group contrasted with the reduced T cell frequency in the duodenal mucosa. This discordance indicates that circulating T cell profiles may not accurately reflect local mucosal immune alterations and highlights the importance of tissue-specific analyses in understanding CVID-associated enteropathy. The opposing patterns may reflect compartment-specific immune regulation, altered T cell trafficking or tissue retention, or local T cell loss. However, these mechanisms were not directly evaluated in the present study and therefore remain speculative.

This study has several limitations. First, the lack of a universally accepted definition of CVID-associated enteropathy and the heterogeneity of this condition represent important limitations in terms of patient classification and between-group comparisons. The exclusion of the two symptomatic patients without histopathological evidence of intestinal inflammation may have increased the immunological contrast between the enteropathy positive and enteropathy negative groups. Because these patients were too few to constitute a separate analytical group, whether symptomatic, histology-negative patients exhibit an intermediate mucosal immune phenotype could not be determined and should be investigated in larger studies. Additionally, tissue sampling limitations should be acknowledged. Histological features of CVID enteropathy may vary across different intestinal regions. Therefore, the use of blind biopsies, as well as sampling restricted to the duodenum and ileum without inclusion of colonic or gastric mucosa, might not fully capture the overall disease spectrum. Given that enteric inflammation is multifactorial and microbial dysbiosis plays a key role, the absence of microbiota analysis represents another limitation of our study.

In conclusion, this study provides a comprehensive tissue-level characterization of CVID-associated enteropathy. Systematically defining immune cell subset profiles and cytokine secretion patterns at both the duodenal and ileal levels, it offers valuable insights into the mucosal immunopathology of the disease. Duodenal T cell profiling revealed that enteropathy positive patients exhibited significantly reduced total T, cytotoxic T, mucosal T, mucosal cytotoxic T, accompanied by an increase in γδ T cells and a marked decrease in intracellular IL-17 levels within mucosal T and γδ T cell subsets. Duodenal analyses demonstrated a trend toward reduced Treg frequencies, whereas ileal Treg frequencies were significantly reduced in the enteropathy positive group relative to healthy controls. Ileal analyses also showed increased γδ T cells and elevated intracellular IFN-γ production by γδ T cells. These findings provide further evidence that altered mucosal T cell regulation, characterized by reduced Treg frequencies, impaired IL-17 responses, and expansion of IFN-γ-producing γδ T cells, may contribute to the pathogenesis of CVID-associated enteropathy. Moreover, these findings may provide a rationale for the development of future IL-17–enhancing therapeutic strategies. The immune profiles and mechanisms elucidated in this study also establish a framework for future mechanistic and translational studies in CVID enteropathy.

## Data Availability

The raw data supporting the conclusions of this article will be made available by the authors, without undue reservation.

## Supplementary material

Supplementary Figure 1 Representative flow cytometry gating strategy. Lymphocytes were first identified based on FSC/SSC characteristics, followed by gating of total T cells (CD3^+^). CD3^+^ cells were subsequently subdivided into T_H_ (CD3^+^CD4^+^), T_C_ (CD3^+^CD8^+^), γδ T cells (CD3^+^TCRγδ^+^), and mucosal T (MT) cells (CD3^+^CD103^+^). MT cells were further classified into MT_H_ (CD3^+^CD103^+^CD4^+^) and MT_C_ (CD3^+^CD103^+^CD8^+^) subsets. Regulatory T cells were identified as CD3^+^CD4^+^CD25^high^FoxP3^+^ cells. SSC, Side scatter, FSC, Forward scatter, T_C_, Cytotoxic T, T_H_, Helper T, Treg, Regulatory T, γδ T, Gamma delta T.

Supplementary Figure 2 Determination of maturation profiles of total and mucosal T cells based on CD45RA and CD27 expression. Naive (CD45RA^+^CD27^+^), central memory (T_CM_; CD45RA^−^CD27^+^), effector memory (T_EM_; CD45RA^−^CD27^−^), and terminally differentiated effector memory (T_EMRA_; CD45RA^+^CD27^−^) subsets were identified. Maturation profiles were analyzed separately in CD8^+^ and CD4^+^ mucosal T cell populations.

Supplementary Figure 3 Representative flow cytometry gating strategy illustrating IL-17 production in mucosal T cells and γδ T cells. Lymphocytes were initially identified based on FSC/SSC characteristics, followed by selection of CD3^+^ T cells. γδ T cells were defined as CD3^+^TCRγδ^+^ cells, and IL-17A expression was subsequently assessed. In parallel, mucosal T cells were identified based on CD103 expression, and intracellular IL-17A production was evaluated within this subset. The plots shown are representative examples demonstrating the gating approach used to quantify IL-17–producing mucosal T cells and γδ T cells. FSC, Forward scatter, SSC, side scatter, TCR, T cell receptor, γδ T, Gamma delta T.
