# Supplementary material for: Mucosal regulatory and effector T-cell imbalance in CVID-associated enteropathy
Source: Front Immunol. 2026 Sep 3;17:1937502. doi: 10.3389/fimmu.2026.1937502 (PMC13582527; doi:10.3389/fimmu.2026.1937502)
Supplement: Supplementary file 1 [file Table1.docx]

SUPPLEMENTARY TABLE 1: Intracellular cytokine expression in duodenal T cell subsets across the study groups [Data are presented as % and median (IQR) for IFN-γ, IL-12, IL-4, IL-17 and IL-10 expression in the evaluated T cell subsets of CVID patients with enteropathy (EP), CVID patients without enteropathy (EN), and healthy controls (HC).]

|  | **EP** | **EN** | **HC** | **p** |
| --- | --- | --- | --- | --- |
| **IFN-γ** |  |  |  |  |
| T_H_ | 15.53 (6.03-20.9) | 17.72 (9.53-23.6) | 24.8 (16.15-27.3) | 0.760 |
| T_C_ | 17.2 (2.82-27.9) | 19.11 (13.6-22.6) | 17.2 (14.1-33.02) | 0.947 |
| Mucosal T | 18.6 (2.85-30.6) | 19.2 (14.3-23.8) | 18.2 (15.2-36.3) | 0.110 |
| Mucosal T_H_ | 17.9 (5.2-22.75) | 19.6 (12.22-23.03) | 20.6 (16.4-31.6) | 0.947 |
| Mucosal T_C_ | 19.1 (2.5-37.8) | 19.38 (13.5-23.8) | 17.25 (14.5-38.5) | 0.991 |
| γδ T | 11.14 (4.0-27.84) | 20.2 (12.75-27.9) | 20.7 (10.9-59.03) | 0.312 |
| **IL-12** |  |  |  |  |
| T_H_ | 11.0 (8.38-15.8) | 11.1 (9.3-16.2) | 12.5 (9.62-14.8) | 0.985 |
| T_C_ | 12.25 (9.1-17.24) | 10.4 (6.3-14.41) | 12.33 (11.8-15.9) | 0.453 |
| Mucosal T | 10.23 (7.6-12.57) | 10.66 (7.1-13.8) | 11.1 (10.8-14.65) | 0.638 |
| Mucosal T_H_ | 11.3 (8.9-23.6) | 13.6 (10.4-17.7) | 11.6 (9.9-13.9) | 0.900 |
| Mucosal T_C_ | 12.02 (8.6-14.8) | 8.6 (5.86-14.5) | 12.4 (11.2-15.45) | 0.594 |
| γδ T | 18.5 (12.8-23.7) | 24.4 (22.14-30.12) | 20.8 (18.2-24.3) | 0.640 |
| **IL-4** |  |  |  |  |
| T_H_ | 6.7 (5.54-10.5) | 6.5 (2.4-12.73) | 9.95 (6.5-24.5) | 0.374 |
| T_C_ | 5.65 (3.3-10.2) | 6.9 (3.5-13.5) | 11.4 (7.9-22.75) | 0.138 |
| Mucosal T | 9.11 (5.26-14.3) | 7.9 (3.6-14.97) | 11.39 (7.6-30.0) | 0.178 |
| Mucosal T_H_ | 7.21 (5.5-11.6) | 7.7 (2.8-14.3) | 11.6 (6.7-30.6) | 0.226 |
| Mucosal T_C_ | 6.74 (4.3-16.3) | 7.64 (3.7-14.8) | 11.5 (7.9-26.54) | 0.417 |
| γδ T | 8.61 (3.8-12.42) | 11.2 (3.2-23.5) | 13.2 (8.3-36.6) | 0.421 |
| **IL-17** |  |  |  |  |
| T_H_ | 10.31 (6.0-19.18) | 20.31 (13.42-42.8) | 18.9 (15.5-55.13) | 0.189 |
| T_C_ | 13.14 (7.65-23.2) | 27.7 (13.92-48.0) | 20.01 (17.14-57.6) | 0.172 |
| Mucosal T | 11.62 (7.2-16.84) | 26.61 (14.3-47.1) | 22.55 (16.7-57.8) | 0.027 |
| Mucosal T_H_ | 13.43 (8.8-19.9) | 22.9 (14.9-46.02) | 21.24 (15.46-55.6) | 0.162 |
| Mucosal T_C_ | 14.27 (7.47-24.03) | 28.5 (14.2-47.2) | 22.37 (17.1-59.1) | 0.249 |
| γδ T | 14.5 (8.9-26.3) | 55.27 (28.9-77.4) | 35.67 (29.9-64.6) | 0.006 |
| **IL-10** |  |  |  |  |
| T_H_ | 15.97 (12.9-21.25) | 18.14 (14.0-29.5) | 23.45 (13.8-30.5) | 0.367 |
| T_C_ | 15.96 (12.9-20.4) | 16.6 (12.02-28.5) | 25.35 (12.9-28.9) | 0.927 |
| Mucosal T | 16.6 (13.6-20.9) | 16.34 (11.5-28.9) | 25.54 (13.3-28.9) | 0.678 |
| Mucosal T_H_ | 16.8 (11.33-21.53) | 16.45 (12.3-29.65) | 24.7 (13.25-28.7) | 0.704 |
| Mucosal T_C_ | 16.2 (12.9-20.8) | 17.02 (11.1-28.7) | 255 (13.25-28.7) | 0.654 |
| γδ T | 17.27 (9.76-23.8) | 12.7 (11.9-35.4) | 24.8 (13.7-32.51) | 0.926 |
| Treg | 30.38 (22-54.6) | 35.9 (18.7-57.9) | 27.77 (16.27-42.9) | 0.982 |
